# The heterogeneity statistic *I*^2^ can be biased in small meta-analyses

**DOI:** 10.1186/s12874-015-0024-z

**Published:** 2015-04-14

**Authors:** Paul T von Hippel

**Affiliations:** Center for Health and Social Policy, LBJ School of Public Affairs, University of Texas, Austin, 2315 Red River, Box Y, Austin, TX 78712 USA

**Keywords:** Meta-analysis, Heterogeneity, Bias

## Abstract

**Background:**

Estimated effects vary across studies, partly because of random sampling error and partly because of heterogeneity. In meta-analysis, the fraction of variance that is due to heterogeneity is estimated by the statistic *I*^2^. We calculate the bias of *I*^2^, focusing on the situation where the number of studies in the meta-analysis is small. Small meta-analyses are common; in the Cochrane Library, the median number of studies per meta-analysis is 7 or fewer.

**Methods:**

We use Mathematica software to calculate the expectation and bias of *I*^2^.

**Results:**

*I*^2^ has a substantial bias when the number of studies is small. The bias is positive when the true fraction of heterogeneity is small, but the bias is typically negative when the true fraction of heterogeneity is large. For example, with 7 studies and no true heterogeneity, *I*^2^ will overestimate heterogeneity by an average of 12 percentage points, but with 7 studies and 80 percent true heterogeneity, *I*^2^ can underestimate heterogeneity by an average of 28 percentage points. Biases of 12–28 percentage points are not trivial when one considers that, in the Cochrane Library, the median *I*^2^ estimate is 21 percent.

**Conclusions:**

The point estimate *I*^2^ should be interpreted cautiously when a meta-analysis has few studies. In small meta-analyses, confidence intervals should supplement or replace the biased point estimate *I*^2^.

## Background

When different studies estimate the effect of a treatment or exposure, the estimates will vary from one study to another. Some of this between-study variance comes from random sampling error, while some may come from *heterogeneity*. There are several sources of heterogeneity, including differences in the treatment, the treated population, the study design, or the data analysis method. When there is no heterogeneity, estimates are said to be *homogeneous* and differ only because of random sampling error.

Heterogeneity is very important. If the existing studies of a treatment are homogeneous, or nearly homogeneous, then there is some assurance that the treatment will have a similar effect when applied to new subjects. On the other hand, if the existing studies are very heterogeneous, then unless the reasons for heterogeneity are well understood, the effect of the treatment on new subjects will be hard to predict [1].

Unfortunately, when studies are compared in a meta-analysis, it is often difficult to say anything definitive about heterogeneity. The reason for this difficulty is that most meta-analyses are small. One summary of the Cochrane Library reported that the median number of studies per meta-analysis was 7 [2], another summary reported that the median was 6 [3], and another reported that the median was just 3 [3]. With so few studies, the classical test for heterogeneity, Cochran’s *Q* [4], is not very informative because its result is as much a function of the number of studies as it is of the amount of heterogeneity. When the number of studies is large, *Q* will often reject the null hypothesis even if the true extent of heterogeneity is trivial, but if the number of studies is small, *Q* provides little power to reject the null hypothesis of homogeneity even if substantial heterogeneity is present [5]. The power of *Q* and other homogeneity tests is further reduced when the studies in the meta-analysis are unbalanced in size—for example, if one of the studies in the meta-analysis is much larger than the others [5].

To better describe heterogeneity, Higgins and Thompson [6] introduced the *I*^2^ statistic, which was meant to improve in two ways on Cochran’s *Q*. First, *I*^2^ is more interpretable than *Q*; specifically, *I*^2^ estimates the proportion of the variance in study estimates that is due to heterogeneity. Second, unlike *Q*, *I*^2^ was meant to be independent of the number of studies; regardless of the number of studies, *I*^2^ ranges from 0 to 1 because it estimates a proportion. The *I*^2^ statistic is now used not just in meta-analysis but also in other analyses where we want to know what fraction of the variance in a set of estimates is due to heterogeneity [7-9].

*I*^2^ does not eliminate the uncertainty that comes from having a small number of studies. No statistic can. In small meta-analyses, for the same reason that *Q* has low power, *I*^2^ is very imprecise. For example, if *Q* fails to reject the null hypothesis of homogeneity, then the confidence interval around *I*^2^ will usually include 0. In meta-analyses from the Cochrane Library, the 95% confidence interval around *I*^2^ typically runs approximately from 0 to .60, implying that up to 60% of the between-study variance could be due to heterogeneity, or there could be no heterogeneity at all [2]. This is not a very informative conclusion. Unfortunately, the uncertainty of the *I*^2^estimate is not obvious to the typical reader of a meta-analysis published in, for example, *Epidemiology* [10,11], the *American Journal of Epidemiology* [12,13], or the Cochrane Library [14]. These outlets do not report the confidence interval around *I*^2^; they only report the point estimate *I*^2^, which may give a false impression of precision.

In this note, we show that *I*^2^ is not just imprecise; it is also biased. Depending on the circumstances, the bias of *I*^2^ can be small or large, positive or negative, but the bias is largest when the number of studies is small and the true fraction of variance that is due to heterogeneity is either very large or very small. For example, in meta-analyses with 7 studies and no true heterogeneity, the *I*^2^ statistic will on average lead us to believe that heterogeneity accounts for about 12% of the between-study variance. At the other extreme, with 7 studies and 80% of the variance due to heterogeneity, the *I*^2^ statistic can on average lead us to believe that just 52% of the variance is due to heterogeneity. These biases of 12 to 28 percentage points are not trivial when one considers that, in the Cochrane Library, the median *I*^2^ value is just 21% [2].

In the following sections, we calculate and illustrate the bias of *I*^2^ and discuss implications for the statistics reported in meta-analyses.

## Methods

We use Mathematica software, version 8, to calculate the expectation and bias of *I*^2^ analytically. This Methods section introduces notation, assumptions, and statistical properties, and describes the calculations that we submitted to Mathematica. The Results section will give the results of those calculations.

### Meta-analysis

Meta-analysis summarizes the results of *K* studies, each of which has sample size *n*_*k*_, *k* = 1,…,*K*. In each study, there is a true effect *β*_*k*_ estimated by ${\widehat{\beta}}_k$, with a true standard error *σ*_*k*_ estimated by ${\widehat{\sigma}}_k$, or, equivalently, a true variance ${\sigma}_k^2$ estimated by ${\widehat{\sigma}}_k^2$. With large *n*_*k*_, the quantity $\left({\widehat{\beta}}_k-{\beta}_k\right)/{\widehat{\sigma}}_k$ approaches a standard normal distribution according to the central limit theorem.

Two models can be used in meta-analysis: a *fixed-effects* model and a *random-effects* model. Some confusion is possible because the term fixed effects is used in two different senses [15]. In some literature, the term fixed effects means that the *K* study effects *β*_*k*_ are assumed to be homogeneous. We use the term fixed effects in its other sense, where it means that we seek only to generalize about the *K* studies in the meta-analysis. The true effects *β*_*k*_ can be either homogeneous or heterogeneous, but they are regarded as fixed quantities. Because of sampling error, the *K* studies would produce different estimates ${\widehat{\beta}}_k$ and ${\widehat{\sigma}}_k$ if they were repeated, but the true effects *β*_*k*_ and true standard errors *σ*_*k*_ would not change.

Under a random-effects model, by contrast, we assume that the true effects *β*_*k*_ in the meta-analysis were drawn at random from a larger population of effects, and we seek to make inferences about that larger population [16]. So the *β*_*k*_ are not fixed quantities but random variables that would be different if a different sample were drawn from the population of effects.

### The estimand *ι*^2^

In order to understand the properties of the estimator *I*^2^, we must first define the quantity that is being estimated. We call the estimand *ι*^2^. It represents the fraction of variance in the estimated effects ${\widehat{\beta}}_k$ that is due to heterogeneity rather than measurement error.

More formally, the ${\widehat{\beta}}_k$ vary from one study to another. The variance in ${\widehat{\beta}}_k$ is partly due to the heterogeneity of the true effects *β*_*k*_ and partly due to estimation error summarized by the standard errors *σ*_*k*_. By the law of total variance we have

1 $$ \begin{array}{c}V\left({\widehat{\beta}}_k\right)=V\left({\beta}_k\right)+E\left({\sigma}_k^2\right)\\ {}={\tau}^2+{\sigma}^2\end{array} $$

where *τ*^2^ = *V*(*β*_*k*_) is the heterogeneity variance or between-study variance, and ${\sigma}^2=E\left({\sigma}_k^2\right)$ is the average within-study variance. Under a fixed-effects model these variances and expectations refer only to the *K* effects *β*_*k*_ and standard errors *σ*_*k*_ in the meta-analysis. Under a random effects model *τ*^2^ refers to the larger population of effects, but *σ*^2^ still refers only to the *K* standard errors *σ*_*k*_ in the meta-analysis, unless we are willing to regard the *σ*_*k*_ as well as the *β*_*k*_ as samples from a larger population.

The fraction of variance that is due to heterogeneity is

2 $$ {\iota}^2=\frac{V\left({\beta}_k\right)}{V\left({\widehat{\beta}}_k\right)}=\frac{\tau^2}{\tau^2+{\sigma}^2} $$

If *ι*^2^ = 0 then the effects *β*_*k*_ are homogeneous; if *ι*^2^ > 0 then they are heterogeneous.

Note that, unlike some past definitions [6], our definition of *ι*^2^ does not assume equal standard errors *σ*_1_ = *σ*_2_ = … = *σ*_*K*_. Note also that *ι*^2^ is not an absolute measure of heterogeneity. Instead, *τ*^2^ is an absolute measure of heterogeneity, while *ι*^2^ compares *τ*^2^ to *σ*^2^. When the estimation error is small, as it is if *n*_*k*_ is large, then *ι*^2^ can be large even if *τ*^2^ is small [17].

### The naïve estimator ${\widehat{\boldsymbol{\iota}}}^{\mathbf{2}}$

To estimate the fraction *ι*^2^, Higgins and Thompson [6] first derived the naïve estimator

3 $$ {\widehat{\iota}}^2=1-\frac{df}{Q} $$

where *df* = *K*–1, *Q* is Cochran’s *Q* statistic [4]

4 $$ Q={\displaystyle \sum_{k=1}^K}\frac{{\left({\widehat{\beta}}_k-\widehat{\overline{\beta}}\right)}^2}{{\widehat{\sigma}}_k^2} $$

and

5 $$ \widehat{\overline{\beta}}=\frac{{\displaystyle {\sum}_{k=1}^K}{\widehat{\sigma}}_k^{-2}{\widehat{\beta}}_k}{{\displaystyle {\sum}_{k=1}^K}{\widehat{\sigma}}_k^{-2}} $$

is the precision-weighted average of the estimated effects.

The distribution of ${\widehat{\iota}}^2$ depends on the distribution of *Q*. Under homogeneity, with large *n*_*k*_, *Q* has a central chi-square distribution with *df* degrees of freedom.

Under heterogeneity, the large-*n*_*k*_ distribution of *Q* depends on whether we regard the effects as fixed or random. Under a random-effects model, *Q* is distributed like a weighted sum of *K*–1 central ${\chi}_1^2$ variables, where the weights are given by a matrix function of *τ*^2^ and ${\sigma}_k^2$ [18]. If we make the simplifying assumption that all the standard errors are equal (*σ*_*k*_*= σ*) then the weights are all equal to 1 + *τ*^2^/*σ*^2^ [18] or, in our notation (1 − *ι*^2^)^− 1^, so that

6 $$ X=\left(1-{\iota}^2\right)Q $$

has a central chi-square distribution with *df* degrees of freedom [18]. As *ι*^2^ gets small, we converge toward the homogeneous situation where *Q* itself has a central chi-square distribution with *df* degrees of freedom.

Under a fixed-effects model, by contrast, *Q* has a non-central chi-square distribution with *df* degrees of freedom and a non-centrality parameter of [19]

7 $$ \lambda ={\displaystyle \sum_{k=1}^K}\frac{{\left({\beta}_k-\overline{\beta}\right)}^2}{\sigma_k^2} $$

where $\overline{\beta}$ is the precision-weighted mean of the true effects *β*_*k*_. If we make the simplifying assumption that all the standard errors are equal (*σ*_*k*_*= σ*) then the non-centrality parameter reduces to

8 $$ \begin{array}{c}\lambda =\frac{1}{\sigma^2}{\displaystyle \sum_{k=1}^K}{\left({\beta}_k-\overline{\beta}\right)}^2\\ {}=K\frac{\tau^2}{\sigma^2}\\ {}=K\frac{\iota^2}{1-{\iota}^2}\end{array} $$

The last line shows that *λ* is an increasing function of *ι*^2^ and that that *λ* = 0 if *ι*^2^ = 0. So again, as *ι*^2^ gets small, *Q* converges toward the central chi-square distribution that it has under homogeneity.

### The truncated estimator *I*^2^

**Figure 1:**
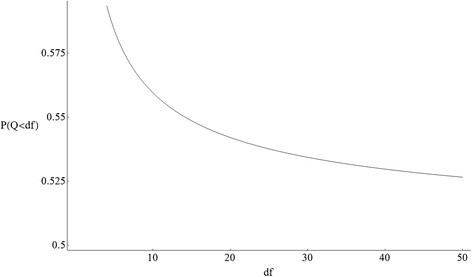
The probability that a central chi-square variable *Q* is less than its degrees of freedom.

A shortcoming of the naïve estimator ${\widehat{\iota}}^2$ is that it can be negative even though the estimand *ι*^2^ cannot. Negative values of ${\widehat{\iota}}^2$ occur whenever *Q* < *df*, which is not a rare event. Figure 1 shows the probability that ${\widehat{\iota}}^2$ is negative when the effects are homogeneous. The probability decreases as *df* increases, but the probability is always greater than 50%.

To avoid negative estimates, Higgins and Thompson [6] suggested rounding them up to zero. The rounded or truncated estimator

9 $$ {I}^2= \max \left(0,{\widehat{\iota}}^2\right) $$

is the estimator that is widely used today. *I*^2^ cannot be negative but can be zero. Values of *I*^2^ = 0 occur in about one-quarter of published meta-analyses [20].

### Expectation and bias of the estimators

The expectation of the naïve estimator ${\widehat{\iota}}^2$ is

10 $$ E\left({\widehat{\iota}}^2\right)=1-df\ E\left(\frac{1}{Q}\right) $$

This is easily calculated in the homogeneous case, where 1/*Q* is an inverse chi-square variable whose expectation is 1/(*df* – 2). It is just as easily calculated in the heterogeneous case with fixed effects; in that case, 1/*Q* is a scaled inverse chi-square variable with an expectation of (1 − *ι*^2^)/(*df* − 2). The calculation is harder in the heterogeneous case with random effects; in that case, 1/*Q* is the scaled inverse of a noncentral chi-square variable. Although the expectation of this inverse has a closed-form solution [21], it is not transparent or easy to calculate by hand. However, we can calculate it using Mathematica.

The expectation of the truncated estimator *I*^2^ is a little harder to calculate. It is the weighted average of two conditional expectations: the expectation of *I*^2^ when *I*^2^ = 0 and the expectation of *I*^2^ when *I*^2^ > 0. The probability that *I*^2^ = 0 is *P*(*Q* < *df*), and the probability that *I*^2^ > 0 is *P*(*Q* > *df*). Therefore the expectation of *I*^2^ is

11 $$ \begin{array}{c}E\left({I}^2\right)=P\left(Q<df\right)\times 0+P\left(Q>df\right)\times E\left({I}^2\left|Q>df\right.\right)\\ {} = P\left(Q>df\right)\times E\left(1-\frac{df}{Q}\left|Q>df\right.\right)\end{array} $$

Under homogeneity, *Q* has a central chi-square distribution and the expectation *E*(*I*^2^) has a closed-form solution which Mathematica can calculate.

Under heterogeneity, the expectation *E*(*I*^2^) depends on whether we regard the effects as fixed or random. If effects are random, then *X* = (1 − *ι*^2^)*Q* has a central chi-square distribution. The probability that *I*^2^ = 0 is *P*(*X* < (1 − *ι*^2^)*df*), and the probability that *I*^2^ > 0 is *P*(*X* > (1 − *ι*^2^)*df*). Therefore the expectation of *I*^2^ is

12 $$ E\left({I}^2\right) = P\left(X>\left(1-{\iota}^2\right)df\right)\times E\left(1-\left(1-{\iota}^2\right)\frac{df}{X}\left|X>\left(1-{\iota}^2\right)df\right.\right) $$

which again has a closed-form solution which Mathematica can calculate.

If instead effects are fixed, then the expectation *E*(*I*^2^) in (11) has no closed-form solution. But the expectation for specific values of *ι*^2^ and *df* can be calculated using numerical integration in Mathematica.

## Results and discussion

### Expectation and bias of *I*^2^ under homogeneity

Under homogeneity, there are two sources of bias in *I*^2^, one positive and one negative. The positive source is larger, so the net bias in *I*^2^ is positive.

The first source of bias is negative bias in the naïve estimator ${\widehat{\iota}}^2=1-df/Q$. Since the estimand *ι*^2^ is zero, the bias of ${\widehat{\iota}}^2$ is the expectation

13 $$ Bias\left({\widehat{\iota}}^2\right)=E\left({\widehat{\iota}}^2\right)=\frac{-2}{df-2} $$

which is negative, and larger if *df* is small.

The second source of bias arises when ${\widehat{\iota}}^2$ is truncated to yield ${I}^2= \max \left(0,{\widehat{\iota}}^2\right).$ Since truncation rounds negative values up to 0, the resulting truncation bias is positive. When *df* is small, truncation is more common (Figure 1), so the truncation bias is more severe.

While this intuitive explanation is helpful, it does not tell us whether the positive and negative components combine to produce a net bias that is positive or negative, large or small. To answer that question, we evaluate the expectation *E*(*I*^2^) in (11), which is also the bias since the estimand is *ι*^2^ = 0. Mathematica gives the bias as

14 $$ Bias\left({I}^2\right)=E\left({I}^2\right)=\left(\frac{df}{df-2}\right)\frac{{\left(\frac{df}{2\mathrm{e}}\right)}^{df/2}-\Gamma \left(\frac{df}{2},\frac{df}{2}\right)}{\Gamma \left(\frac{df}{2}+1\right)} $$

where *Γ*(*df*/2 + 1) is the gamma function and *Γ*(*df*/2, *df*/2) is the upper incomplete gamma function (which has two arguments).

**Figure 2:**
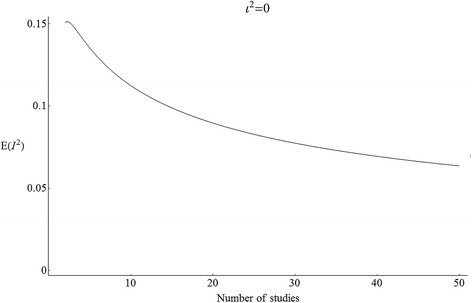
The expectation of *I* ^2^ when *ι* ^2^ = 0.

It is hard to tell by inspecting (14) whether the bias is positive or negative, small or large. To visualize the answer, Figure 2 plots the expectation *E*(*I*^2^), which is also the *Bias*(*I*^2^), as a function of the number of studies *K* = *df* + 1. The bias is always positive, indicating that the positive truncation bias outweighs the negative bias in in ${\widehat{\iota}}^2$. The bias shrinks at a decreasing rate as *K* grows. With *K* = 3 studies (which is the median in one summary of the Cochrane Library [22]), the bias is undefined because *E*(*I*^2^) is only defined if *df* > 2. With *K* = 7 studies (which is the median in another summary of the Cochrane Library [2]), the bias is .12. With *K* = 10 studies, the bias is .11; with *K* = 50 studies the bias is .06.

### Expectation and bias of *I*^2^ under heterogeneity

Under heterogeneity, the expectation *E*(*I*^2^) depends on whether we regard the effects as fixed or random.

#### Random-effects model

With random effects, there are still two sources of bias in *I*^2^, one positive and one negative. But now the positive source can be either smaller or larger than the negative source, so that the overall bias can be either negative or positive.

The first source of bias is negative bias in the naïve estimator ${\widehat{\iota}}^2$:

15 $$ \begin{array}{c} Bias\left({\widehat{\iota}}^2\right)=E\left(1-\frac{df}{Q}\right)-{\iota}^2\\ {}=\frac{2{\iota}^2-2}{df-2}\end{array} $$

This bias is always negative since 0 ≤ *ι*^2^ < 1. The bias is larger if *df* is small.

The second source of bias arises when ${\widehat{\iota}}^2$ is truncated to yield ${I}^2= \max \left(0,{\widehat{\iota}}^2\right).$ Since truncation rounds negative values up to 0, truncation yields a positive bias. The truncation bias is smaller if *df* is large or *ι*^2^ is large. This is because the probability of truncation is a little smaller when *df* is large, and a lot smaller when *ι*^2^ is large. (From (12) the probability of truncation is *P*(*X* > (1 − *ι*^2^)*df*), where $X\sim {\upchi}_{df}^2$).

Intuitively, when *ι*^2^ is small, we approach the homogeneous case where the bias in *I*^2^ is positive because of truncation. However, when *ι*^2^ is large, truncation is less common and the bias in *I*^2^ approaches the bias of ${\widehat{\iota}}^2$, which is negative.

More formally, under a random-effects model, the expectation *E*(*I*^2^) in (12) has a solution which Mathematica gives as

16 $$ E\left({I}^2\right)=\frac{\left(-2{\mathrm{e}}^{\frac{1}{2}df\left({\iota}^2-1\right)}\left(df\left({\iota}^2-1\right)-2\right)-df\left({\iota}^2-1\right)\left(df{\iota}^2-2\right){E}_{-\frac{\mathrm{df}}{2}}\left(\frac{1}{2}\left(df-df{\iota}^2\right)\right)\right)}{\left(df-2\right)df{E}_{1-\frac{df}{2}}\left(-\frac{1}{2}df\left({\iota}^2-1\right)\right)} $$

where expressions of the form *E*_*n*_(*z*) represent the exponential integral function.

The expectation in (16) is in closed form but is even less transparent than its predecessor in (14). It is not clear from inspection whether the bias is large or small, positive or negative.

**Figure 3:**
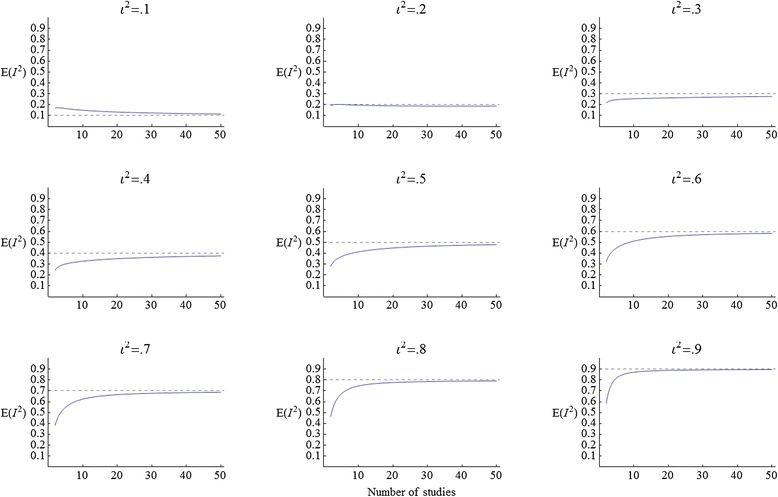
The expectation of *I* ^2^ under a random-effects model, for *ι* ^2^=.1,.2, …,.9.

To visualize *E*(*I*^2^), Figure 3 gives a graphics grid displaying 9 plots of *E*(*I*^2^) as a function of *K*, for *ι*^2^ values between .1 and .9. In each plot, a dotted line is drawn at the value of the estimand *ι*^2^, so that the bias of *I*^2^ is the difference between the dotted line and the curve *E*(*I*^2^).

The bias is generally larger for small *K*. At *ι*^2^ = .1 the bias is positive. At *ι*^2^ = .2 there is practically no bias, and above *ι*^2^ = .2 the bias switches from positive to negative. As *ι*^2^ increases beyond .2 the bias gets larger for small *K*, but smaller for large *K*.

When *K* is large there is practically no bias, particularly if *ι*^2^ is large as well. But when *K* is small, as is often the case in meta-analysis, the bias can be noticeable even if *ι*^2^ is large. For example, if *ι*^2^ = .8 and *K* = 7 (a typical or even high value for the Cochrane Library [2]), the expectation of *I*^2^ is just .52.

#### Fixed-effects model

Under heterogeneity with fixed effects, Mathematica gives the expectation of the naïve estimator ${\widehat{\iota}}^2$ as

17 $$ E\left({\widehat{\iota}}^2\right)=1+df\ {2}^{df/2-2}\ {\mathrm{e}}^{-\lambda /2}{\left(-1\right)}^{-df/2}{\lambda}^{1-df/2}\left(\Gamma \left(\frac{df}{2}-1\right)-\Gamma \left(\frac{df}{2}-1,-\frac{\lambda }{2}\right)\right) $$

where *λ* = *Kι*^2^/(1 − *ι*^2^) from equation (8). However, this expression for $E\left({\widehat{\iota}}^2\right)$ is only real if *df* is even.^a^ If *df* is odd, a much longer exact expression for $E\left({\widehat{\iota}}^2\right)$ can be derived using results in [21], or an approximation can be obtained numerically.

The bias of the naïve estimator is ${\widehat{\iota}}^2-{\iota}^2$. Although it is not obvious from inspection, the bias is negative for *ι* < .8, and very slightly positive for *ι* ≥ .8.

The bias of the truncated estimator *I*^2^ is a little different. Intuitively, when *ι*^2^ is small, we approach the homogeneous case where the bias in *I*^2^ is positive because of truncation. However, as *ι*^2^ gets large, truncation is less common and the bias in *I*^2^ approaches the bias of ${\widehat{\iota}}^2$, which again is negative for *ι* < .8, and very slightly positive for *ι* ≥ .8.

The expectation of the truncated estimator *I*^2^ can be calculated from equation (11) but under a fixed-effects model the solution no longer has a closed form, not even a complicated one. Instead, to evaluate *E*(*I*^2^) we use numerical integration in Mathematica.

**Figure 4:**
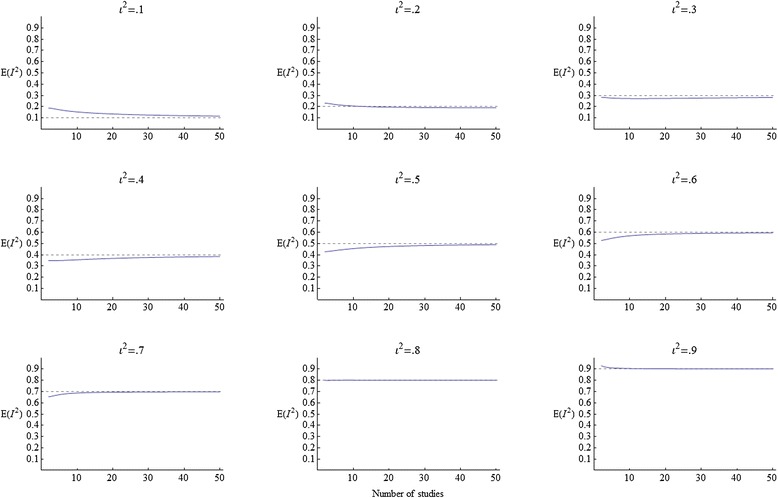
The expectation of *I* ^2^ under a fixed-effects model, for *ι* ^2^=.1,.2, …,.9.

Figure 4 is a graphics grid displaying 9 plots of *E*(*I*^2^) as a function of *K*, for *ι*^2^ values between .1 and .9. The bias is generally larger for small *K*. At *ι*^2^ = .1 the bias is positive. At *ι*^2^ = .2 there is practically no bias except for very small *K*. Above *ι*^2^ = .2 the bias switches from positive to negative. As *ι*^2^ increases from .3 to .5 the negative bias gets larger, but as *ι*^2^ increases further from .6 to .7, the bias gets smaller and is increasingly restricted to small values of *K*, until at *ι*^2^ =.8 there is practically no bias. At *ι*^2^ =.9 the bias is positive again but very small and restricted to very small values of *K*.

In general, the bias is milder under the fixed-effects model than under the random-effects model, particularly if *ι*^2^ is large. For example, if *ι*^2^ = .8 and *K* = 7 (a typical or even high value for the Cochrane Library [2]), the expectation of *I*^2^ is just .52 under the random-effects model but is .80 (practically unbiased) under the fixed-effects model.

## Conclusions

We have shown that, in small meta-analyses, the widely used heterogeneity statistic *I*^2^, which was already known to be imprecise, is biased as well. The bias shrinks as the number of studies *K* grows, but since *K* is often small in published meta-analyses, the bias of *I*^2^ is often large in practice.

The bias and imprecision of *I*^2^ are to some extent unavoidable and should not be taken as a criticism of the *I*^2^ statistic itself. All statistics are imprecise in small samples, and any reasonable estimator of the heterogeneity fraction *ι*^2^ will be biased when the true value of *ι*^2^ is close to 0. The reason for the bias is fundamental. Like the estimand *ι*^2^, any reasonable estimator should be limited to nonnegative estimates, but the expectation of those nonnegative estimates will be positive and will exceed *ι*^2^ when the true value of *ι*^2^ is close to 0.

Similar bias has been observed in the heterogeneity variance *τ*^2^. Any reasonable estimator of *τ*^2^ will be limited to nonnegative values, and this will cause bias when the true value of *τ*^2^ is close to zero [15,23]. Estimators of *τ*^2^ have been constructed that are less biased or more precise under some circumstances, but all nonnegative estimators are biased when the true value of *τ*^2^ is close to zero [24].

Despite its bias and imprecision, the *I*^2^ statistic remains useful. In large meta-analyses, *I*^2^ can be precise with little bias, and even in small meta-analyses it is better to have a biased and imprecise estimate of *ι*^2^ than it is to have no estimate at all. In addition, although the bias of *I*^2^ depends to some extent on the number of studies *K*, *I*^2^ is much less dependent on *K* than *Q* is.

Nevertheless, *I*^2^ should be presented and interpreted cautiously in small meta-analyses. Perhaps the most straightforward response to the bias and imprecision of *I*^2^ is to report a 95% confidence interval in addition to—or even instead of—the point estimate *I*^2^. Although methods for calculating confidence intervals around *I*^2^ can be a bit complicated [6,19,23,25], the best methods have good coverage and they give a sense of the range of possible *ι*^2^ values without highlighting a point estimate that may be biased and imprecise. While some meta-analyses do report confidence intervals around *I*^2^ [26], such confidence intervals are not included in recent meta-analysis published in journals such as *Epidemiology* [10,11], the *American Journal of Epidemiology* [12,13]*,* or the Cochrane Library. Journals publishing meta-analysis should consider requiring confidence intervals for *ι*^2^.

In small meta-analyses, confidence intervals for *ι*^2^ are often very wide [2] but their width tells us something. The width of the confidence intervals tells us how little information a small meta-analysis typically provides about heterogeneity. In many small meta-analyses, we may not be able to estimate heterogeneity with much precision; in fact, we may have little confidence in any estimate beyond the average effect size. No statistic can change the limitations of small meta-analyses, and the statistics that we report should make those limitations clear.

## Endnote

^a^We filed a bug report with Wolfram Research regarding Mathematica’s failure to provide a real solution for odd *df*.
